# Unmasking Dual Vascular Complications of Sunitinib in Advanced Renal Cell Carcinoma

**DOI:** 10.7759/cureus.95523

**Published:** 2025-10-27

**Authors:** Stella Nabirye, Doreen Nakagaayi, Wanzhu Zhang, Emmy Okello

**Affiliations:** 1 Adult Cardiology, Uganda Heart Institute, Kampala, UGA; 2 Internal Medicine, Kiruddu National Referral Hospital, Kampala, UGA; 3 Internal Medicine, Makerere University College of Health Sciences, Kampala, UGA

**Keywords:** acute myocardial infarction, arterial thromboembolism, renal cell caricnoma, tyrosine kinase inhibitors (tki), venous thromboembolsim

## Abstract

Tyrosine kinase inhibitors (TKIs) such as sunitinib have transformed the management of advanced renal cell carcinoma (RCC), yet their association with thromboembolic complications remains incompletely understood. Arterial events, including myocardial infarction, are rare but clinically significant and pose a management dilemma.

We report the case of a male in his mid-thirties, a sickle cell carrier, diagnosed with metastatic renal cell carcinoma. Following radical nephrectomy, he commenced sunitinib-based chemotherapy. Within months, he developed extensive upper extremity venous thromboses involving the left internal jugular, left subclavian, and left axillary, and, subsequently, an acute ST-elevation myocardial infarction (STEMI), despite being on anticoagulation. Coronary angiogram revealed thrombotic occlusion of the mid-left descending artery, successfully managed with percutaneous coronary intervention.

This case highlights the convergence of venous and arterial thrombosis in a patient with RCC receiving TKI therapy, underscoring the potential for life-threatening vascular complications associated with sunitinib, especially in patients with additional prothrombotic predispositions. The elevated thromboembolic risk among patients with metastatic RCC on sunitinib calls for high-quality clinical vigilance. Early recognition, individualized thrombotic risk assessment, and timely intervention are essential for optimizing patient safety and reducing morbidity and mortality during TKI therapy.

## Introduction

Renal cell carcinoma (RCC) accounts for approximately 2-3% of all adult malignancies, with an increasing incidence globally [1]. Targeted anti-cancer therapies, such as multi-targeted tyrosine kinase inhibitors (TKIs) like sunitinib and sorafenib, and also immunomodulators, have become frontline agents in the management of pancreatic, gastrointestinal stromal tumours, and metastatic renal cell carcinoma [2]. However, TKIs have been associated with cardiovascular toxicity, including venous and arterial thrombosis, hypertension, aneurysms, arterial dissections, myocardial infarction, pericardial effusion, and arrhythmias, occurring in approximately 10% of individuals [3]. The incidence of arterial thrombosis among patients of TKIs has been reported to be 1.4% [4]. The risk of venous thromboembolism (VTE) has been noted to be highest after the diagnosis of cancer, with the relative risk of developing venous thromboembolism in cancer of the kidney observed to be 3.4 ( 95% CI: 1.9-6.2) [5]. While VTE is a recognized complication in oncology, the occurrence of arterial events such as acute myocardial infarction (AMI) remains rare and underreported [6]. When both venous and arterial thromboses occur in close succession, particularly in a patient with additional prothrombotic

## Case presentation

We present a rare case of a male in his mid-thirties with sickle cell trait, a non-smoker, with occasional alcohol use. He had no known food or drug allergies and was married with two children. His initial complaint was a seven-year history of chronic back pain managed intermittently with analgesics. Seven months before admission to our facility, he presented to another health facility with painless gross haematuria unresponsive to steroids and tranexamic acid. Urinalysis revealed red blood cells, and a pelvic ultrasound showed an enlarged prostate. He subsequently underwent a contrast-enhanced abdominal computer tomography scan, which identified a left renal mass with lumbosacral and lumbar vertebral metastases, suggestive of renal cell carcinoma (RCC) staged as TXN0M1.

A multidisciplinary team recommended total radical nephrectomy, which was performed a month later after the first presentation. Histopathology confirmed renal medullary carcinoma. He was referred to oncology and commenced on a tyrosine kinase inhibitor (TKI) (sunitinib) and zoledronic acid. Soon after initiating therapy, the patient developed fatigue, palpitations, non-specific chest pain, hypertension, acral hypopigmentation, microaneurysms on the sole, and minor bleeding under nail beds. His sunitinib dose was reduced from 50mg to 37.5mg on alternate days, and telmisartan-hydrochlorothiazide 40/12.5mg once a day.

Four months after surgery and while on chemotherapy, he developed left-sided supraclavicular pain radiating to the left arm, with associated difficulty in breathing. Imaging revealed extensive thrombosis of the left jugular, subclavian, and axillary veins. Rivaroxaban was initiated.

Approximately six months into chemotherapy, he presented with acute, squeezing left-sided chest pain radiating to the left shoulder and arm, accompanied by sweating and shortness of breath. He was referred to our tertiary specialist facility for further management about seven hours after the onset of chest pain.

On arrival, he appeared ill but was afebrile with stable vitals: BP 120/77 mmHg, pulse 74 bpm (regular and synchronous), RR 18 bpm, and SpO₂ 94% on room air. He had no edema, jaundice, dehydration, cyanosis, or lymphadenopathy. Cardiovascular and respiratory exams were unremarkable. Abdominal exam revealed a left lumbar surgical scar without palpable masses or tenderness. Neurological evaluation was normal. Electrocardiography was immediately performed, followed by other investigations like echocardiography and laboratory studies.

**Figure 1:**
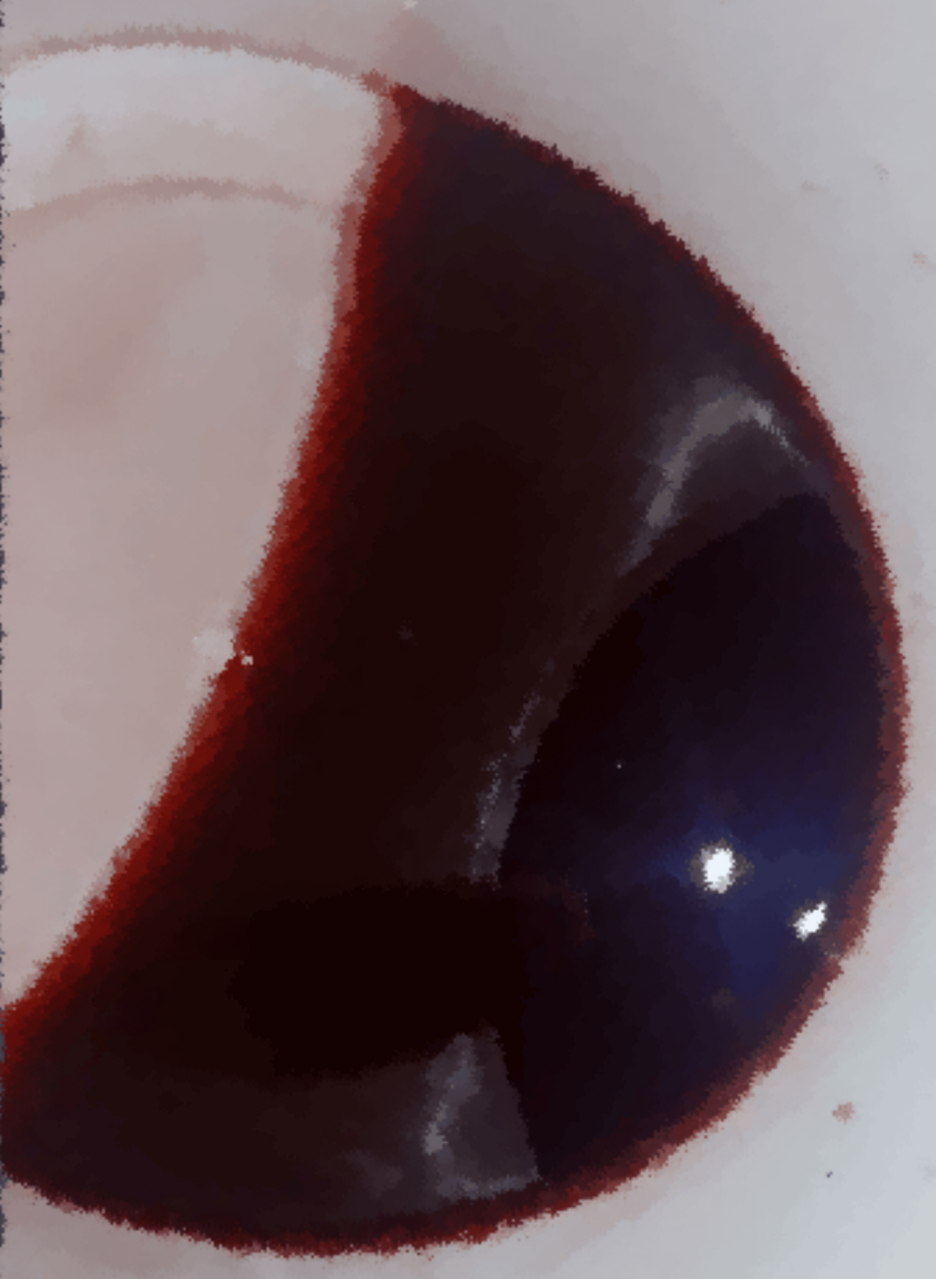
Hematuria of the patient

**Figure 2:**
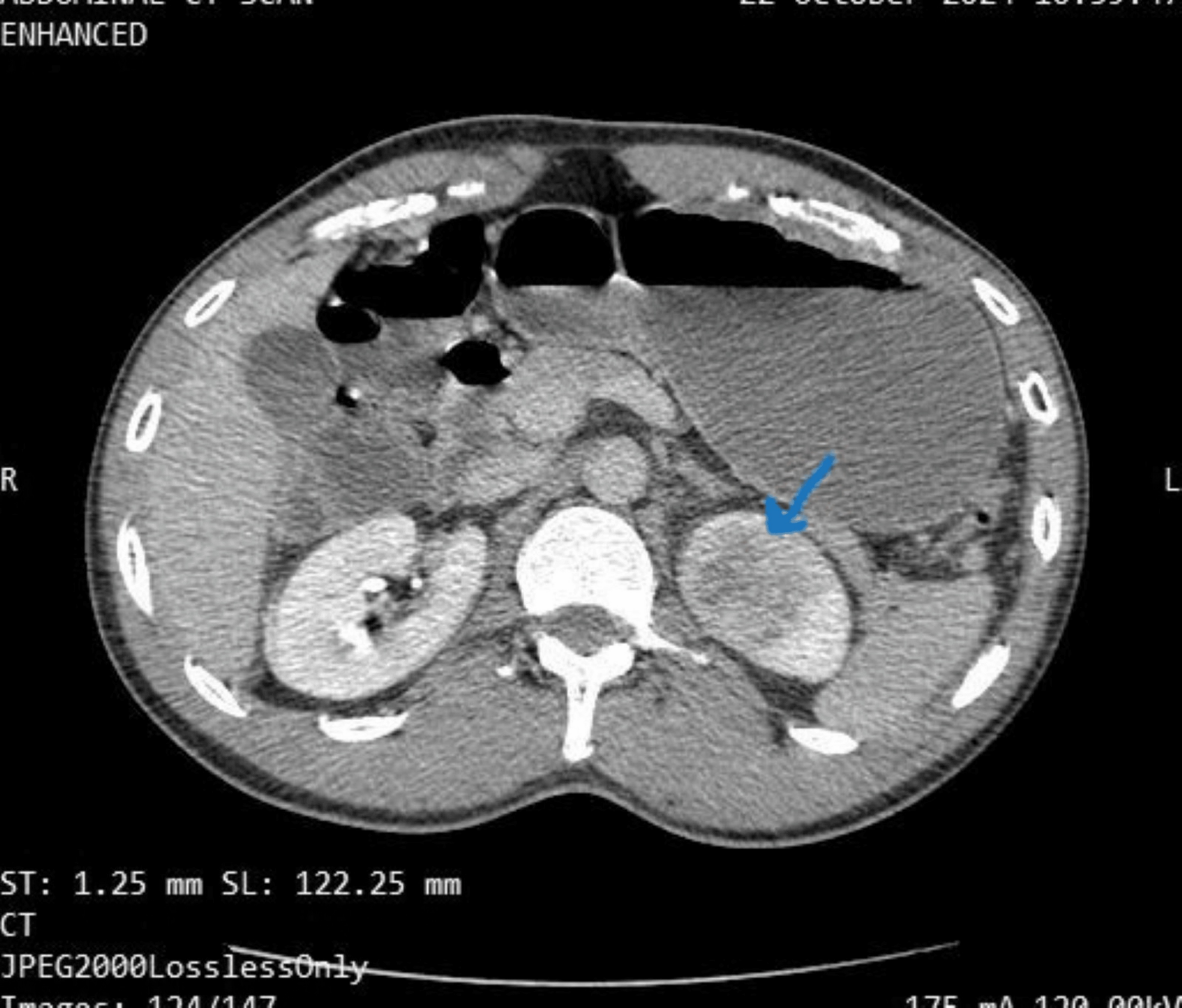
Abdominal computer tomography with contrast showing an ill-defined iso-dense non-enhancing mass in the left kidney

**Figure 3:**
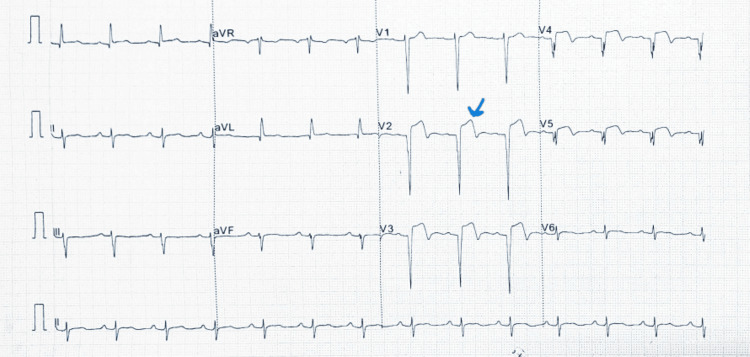
Electrocardiograph showing pathological Q waves and ST segment Elevation in V1-V5

Laboratory studies showed hematuria (Figure 1), elevated creatinine of 130mmol/L (reference range: 63.3-110 mmol/L), troponin of 34.72ng/ml (reference range: 0.02-0.06 ng/ml), D-dimers of 6.43 (reference range: 0.0-1.0 microgram/ml), total cholesterol of 230mg/dl (reference range: 0.0-160.0mg/dl) and LDL 161.9 mg/dl (reference range 0.0-160.0 mg/dl). Abdominal computer tomography with contrast showed an ill-defined iso-dense non-enhancing mass in the left kidney (Figure 2). The electrocardiograph revealed pathological Q-waves and ST-segment elevation in leads V2-V5 (Figure 3). Echocardiography showed mild anteroseptal wall hypokinesia with normal left ventricular systolic function.

The final diagnosis included metastatic renal cell carcinoma (status post total radical nephrectomy), currently on sunitinib, sunitinib-induced hypertension, extensive upper extremity venous thrombosis involving the jugular, subclavian, and axillary veins, and acute anterior ST-elevation myocardial infarction (STEMI).

Management

**Figure 4:**
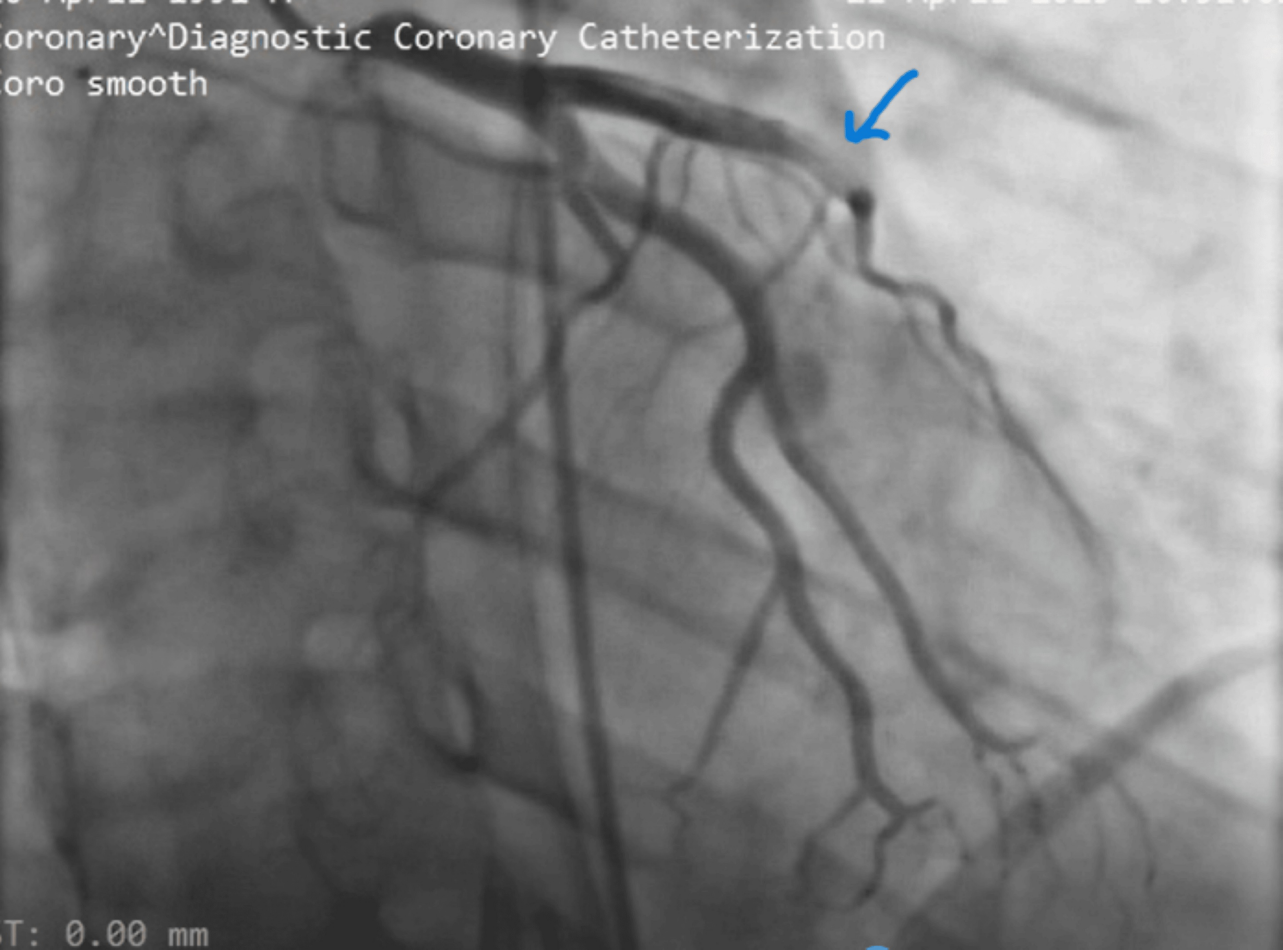
Coronary angiogram showing occlusion of the left coronary system with occlusion of mid left anterior descending artery (arrow)

**Figure 5:**
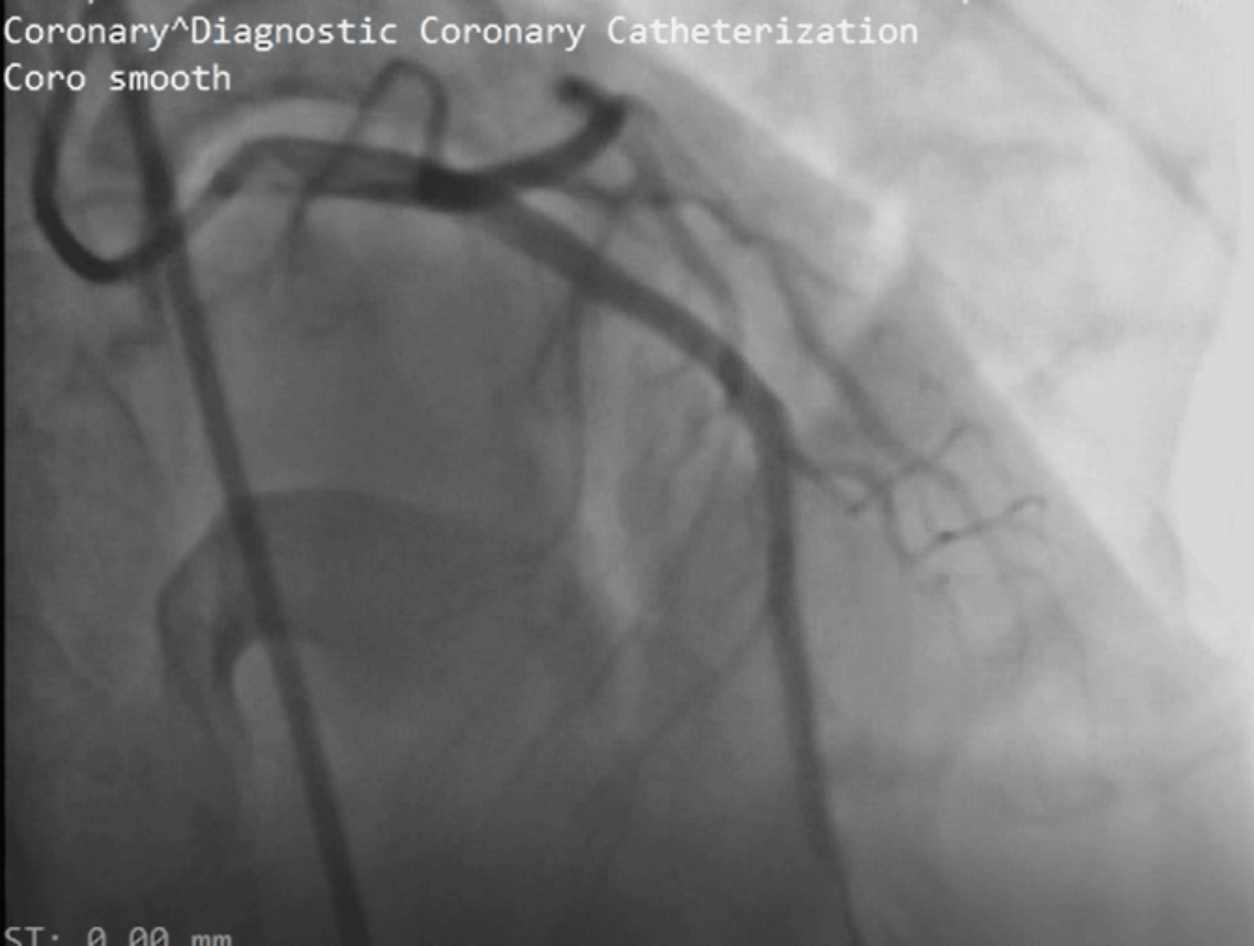
Left coronary system post percutaneous coronary intervention

He received nitroglycerin spray, morphine (later switched to fentanyl), 300mg aspirin, and 300mg clopidogrel. Due to the anticipated delay for primary percutaneous coronary intervention, tenecteplase (40mg) was administered. He developed mild hemoptysis post-lysis, which resolved spontaneously. Beta-blockers and high-dose rosuvastatin were initiated. Twelve hours post-lysis, the coronary angiogram revealed a thrombus in the mid-left anterior descending artery (Figure 4). A successful percutaneous coronary intervention (PCI) with drug-eluting stent placement was performed (Figure 5). This resulted in an improvement of symptoms and ECG changes. He was discharged on triple antithrombotic therapy (clopidogrel, aspirin, and apixaban) for one month due to concurrent venous and arterial thrombus with a stent. Our patient was advised to return to his daily activities after he had improved. Follow-up care after 30 days included cardiology review with de-escalation of triple antithrombotic therapy to apixaban and clopidogrel, and continued oncology care with ongoing chemotherapy. 

## Discussion

This case highlights an uncommon but clinically significant intersection of malignancy, targeted therapy, and systemic thrombotic events. Our patient, a male in his thirties with metastatic renal medullary carcinoma on sunitinib, developed hypertension, extensive VTE, and an acute STEMI, despite receiving anticoagulation. His concurrent sickle cell trait further complicated the thrombotic risk landscape.

The risk of thrombosis in this patient was multifactorial, including metastatic RCC, sunitinib therapy, sickle cell trait, chemotherapy-induced hypertension, and dyslipidaemia. RCC, particularly in its metastatic form, is among the most thrombotic solid tumors [7]. Tumor-driven cytokine release, vascular invasion, and hypercoagulability contribute to a heightened baseline risk for both venous and arterial events. RCC’s angioinvasive nature, combined with reduced mobility and occasional paraneoplastic states, places patients in a sustained prothrombotic state [8].

In this case, the risk was likely amplified by the initiation of sunitinib, a TKI with known anti-angiogenic effects. Sunitinib's inhibition of vascular endothelial growth factor (VEGF) and platelet-derived growth factor (PDGF) signalling impairs endothelial integrity, disrupts vascular repair, and induces hypertension, all of which predispose to both arterial thrombosis and VTE [9]. While VTE is more commonly reported with TKIs, arterial thromboses, especially myocardial infarction, though rare, are increasingly recognized [10].

Additionally, sickle cell trait could be a subtle risk amplifier. Although sickle cell trait (SCT) is traditionally considered benign, recent literature reveals a modest yet measurable increase in thrombotic risk, particularly in high-risk clinical environments [11, 12]. The presence of SCT has been associated with a 1.5- to 2-fold increased risk of VTE, especially under conditions of endothelial stress, inflammation, or malignancy [12, 13]. In vitro, SCT erythrocytes exhibit altered deformability and adherence profiles, potentially contributing to vascular stasis and local microthrombosis [14]. In this case, SCT may have acted synergistically with the underlying malignancy and sunitinib therapy to potentiate systemic thrombotic activation.

Navigating the therapeutic crossroads

A key challenge was managing dual thromboses, venous and coronary, in a patient already exhibiting bleeding complications (e.g., post-thrombolysis hemoptysis). The administration of thrombolytic therapy for STEMI, followed by PCI, required escalation to triple antithrombotic therapy (anticoagulation plus dual antiplatelets), a strategy known to increase haemorrhagic risk substantially.

In addition to its prothrombotic effects, sunitinib is associated with an increased risk of bleeding due to its anti-angiogenic properties and potential impairment of platelet function. By inhibiting VEGF and PDGF pathways, sunitinib disrupts vascular integrity and may compromise haemostatic mechanisms, particularly in patients receiving concurrent anticoagulation or antiplatelet therapy [9].

In this case, the development of hemoptysis following thrombolysis necessitated careful reassessment of bleeding risk. The care team adhered to international best practice by limiting triple therapy to one month, selecting a direct oral anticoagulant (DOAC) with a favourable bleeding profile (apixaban), and emphasizing early de-escalation strategies [15]. This case underscores the necessity of individualized thrombotic-bleeding risk balancing in cardio-oncology.

Most reports describe either venous or arterial thromboembolism following chemotherapy, but few document both occurring concurrently. Batra et al reported a young adult with carotid artery thrombus and pulmonary embolism while on cisplatin for testicular cancer [16]. Ahmad et al. described a patient who developed pulmonary artery thrombosis shortly after starting sunitinib, prompting immediate anticoagulation and discontinuation of sunitinib therapy [17].

Matko et al described a 73-year-old patient who developed acute coronary syndrome after nephrectomy and sunitinib initiation, despite having only obesity as the risk factor [18]. Similarly, our patient had no prior coronary artery disease risks but developed hypertension and dyslipidaemia after starting sunitinib, followed by thromboembolism, suggesting a direct cardiotoxic effect. The clinical spectrum of TKI toxicity ranges from mild symptoms such as fatigue and stomatitis to serious cardiovascular complications [2]. Cardiac toxicity may be underestimated, as symptoms can mimic oncologic disease progression. In a study of patients with metastatic RCC, nearly one-third exhibited either biochemical or clinical signs of cardiac toxicity [3]. Early recognition and management, including the use of ACE inhibitors, beta-blockers, and simvastatin, are essential to mitigate cardiovascular risk and support safe continuation of therapy [19].

This case underscores the importance of early cardiovascular risk assessment and monitoring in patients receiving TKIs, especially those with additional prothrombotic predispositions. The concurrent development of VTE and STEMI in a young patient without prior cardiac risk factors highlights the need for heightened clinical vigilance and individualized therapy. Early recognition and timely intervention are essential to improving outcomes in cardio-oncology.

## Conclusions

The coexistence of RCC and chemotherapy represents two swords, with both independently contributing to an elevated risk of thromboembolism. Our case underscores the potential for simultaneous arterial and venous thrombotic events in patients initiated on TKIs, highlighting the need for vigilant surveillance and prompt reporting. This further reinforces the importance of maintaining a low threshold for screening and evaluating patients for thromboembolic complications shortly after initiating agents such as sunitinib. Early detection and intervention may help prevent or mitigate complications, including acute coronary syndrome or venous thrombosis, which are associated with significant morbidity and mortality.
